# The Influence of Resistance Training Experience on the Efficacy of Motor Imagery for Acutely Increasing Corticospinal Excitability

**DOI:** 10.3390/brainsci13121635

**Published:** 2023-11-25

**Authors:** Emily J. Parsowith, Matt S. Stock, Ryan M. Girts, Jonathan P. Beausejour, Ariel Alberto, Joshua C. Carr, Kylie K. Harmon

**Affiliations:** 1 Cognition, Neuroplasticity, Sarcopenia (CNS) Laboratory, Institute of Exercise Physiology and Rehabilitation Science, School of Kinesiology and Rehabilitation Sciences, University of Central Florida, Orlando, FL 32816, USA; emily.parsowith@ucf.edu (E.J.P.); matt.stock@ucf.edu (M.S.S.); jonathan.beausejour@ucf.edu (J.P.B.); 2 Department of Natural and Health Sciences, Pfeiffer University, Misenheimer, NC 28109, USA; ryan.girts@pfeiffer.edu; 3 School of Medicine, Case Western Reserve University, Cleveland, OH 44106, USA; axa1488@case.edu; 4 Department of Kinesiology, Texas Christian University, Fort Worth, TX 76109, USA; joshua.carr@tcu.edu; 5 Department of Medical Education, Anne Burnett Marion School of Medicine at Texas Christian University, Fort Worth, TX 76109, USA; 6 Department of Exercise Science, Syracuse University, Syracuse, NY 13244, USA

**Keywords:** motor imagery, TMS, resistance–training, corticospinal excitability, force, torque, strength, motor cortex, motor unit, MEP

## Abstract

Both motor imagery and resistance–training enhance motor function and corticospinal excitability. We tested the hypothesis that young participants with significant resistance–training experience would show heightened corticospinal excitability during a single session of motor imagery training. Fifty-six participants (mean ± SD age = 22 ± 2 years) were divided into resistance–trained and untrained groups. Forty-one upper-body resistance trained (21 males, 20 females; mean ± SD relative one repetition maximum bench press = 0.922 ± 0.317 kg/kg) and 15 untrained (4 males, 11 females; mean ± SD relative one repetition maximum bench press = 0.566 ± 0.175 kg/kg) participants visited the laboratory on three separate occasions. The first visit served as the familiarization session. During visits 2 and 3, participants engaged in a hand/wrist motor imagery protocol or rested quietly (control condition) in a randomized order. Before and after the interventions, single-pulse transcranial magnetic stimulation (TMS) over the motor cortex was used to measure resting motor–evoked potential amplitude of the first dorsal interosseous muscle. Our main finding was that motor imagery acutely increased corticospinal excitability by ~64% (marginal means pre = 784.1 µV, post = 1246.6 µV; *p* < 0.001, *d* = 0.487). However, there was no evidence that the increase in corticospinal excitability was influenced by resistance–training experience. We suspect that our results may have been influenced by the specific nature of the motor imagery task. Our findings have important implications for motor imagery prescription and suggest that motor imagery training may be equally beneficial for both resistance–trained and untrained populations. This study was prospectively registered at ClinicalTrials.gov (Identifier: NCT03889548).

## 1. Introduction

Regular participation in resistance–training increases maximal strength and force production. It is generally accepted that upon initiating a novel resistance–training program, neural adaptations facilitate the initial, rapid increase in maximal force production, whereas skeletal muscle hypertrophy becomes more prominent after several weeks of training [1,2]. The rapid improvement in maximal force following only a few weeks of training has led some to suggest that there is a large motor skill component to resistance–training [3,4]. Some of the most important neural adaptations to resistance–training include changes in agonist/antagonist coactivation [2], improvements in voluntary activation [5], and alterations in corticospinal excitability and/or inhibition [6]. Studies using transcranial magnetic stimulation (TMS) have reported enhanced excitability of cortical neurons projecting to muscles after several weeks of training [7]. Functional MRI studies have also revealed altered activity in motor areas of the brain, suggestive of neural remodeling due to resistance–training [8]. These neural adaptations may facilitate an increased voluntary drive from the motor cortex to activate motor units more efficiently [9]. The time course and magnitude of these adaptations appear to be specific to the demands of the training program [10]. As such, when cross-sectionally comparing resistance–trained versus untrained individuals, the extent to which the central nervous system has undergone adaptation and remodeling likely explains differences in maximal force generation.

Motor imagery tasks require individuals to imagine that they are voluntarily contracting a target muscle group. Motor imagery has been shown to facilitate neural adaptations in a manner similar to those observed following voluntary activation, providing alternative and complementary means of remodeling the motor system [11]. Motor imagery evokes responses in several brain regions known to control motor function, such as the primary motor cortex, supplementary and premotor areas, and cingulate gyrus [12]. These observations have led investigators to explore the potential of motor imagery to enhance corticospinal excitability in the absence of overt movement [13]. A comparative TMS study designed to quantify differences between motor imagery, action observation, and imitation found that while voluntary contractions produced the greatest increase in corticospinal excitability, the conditions involving no apparent movement produced significant increases as well [14]. Furthermore, previous investigations have shown that motor imagery increases maximal voluntary strength and even attenuates strength loss during periods of wrist immobilization [15,16]. Further evidence of this phenomenon has been demonstrated in studies involving single-limb training, where the strength of the contralateral untrained muscle improved in the experimental groups that utilized motor imagery training [14,15]. Thus, activation of different brain regions related to motor control during motor imagery may promote neural adaptation to resistance–training.

Previous motor imagery investigations have studied healthy adults with vaguely defined resistance–training experience or patients with neuromuscular disorders. While both resistance–training and motor imagery have been shown to enhance maximal strength and corticospinal adaptations [5,10,11,15], the extent to which resistance–trained versus untrained participants uniquely respond to motor imagery is unknown. Understanding the extent to which previous resistance–training experience influences the responsiveness to motor imagery has important implications for the development of targeted neural and rehabilitation interventions. Therefore, the purpose of this study was to examine whether resistance–training experience results in differences in corticospinal excitability during a motor imagery task. We hypothesized that performing a motor imagery task would increase corticospinal excitability, but greater improvements would be observed among participants with resistance–training experience due to their previous neural adaptations derived from the performance of repetitive, high–force contractions.

## 2. Materials and Methods

### 2.1. Experimental Approach to the Problem

**Figure 1:**
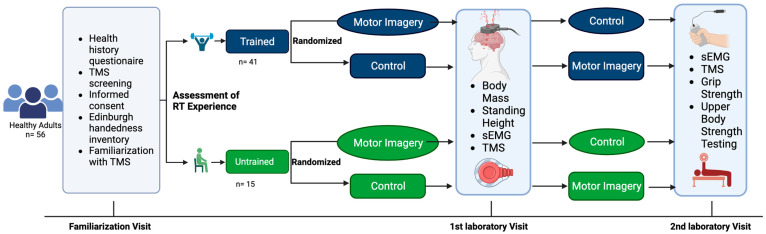
An overview of the experimental design. Created using Biorender.com (accessed on 21 October 2023).

This study utilized a repeated measures design in healthy adults between the ages of 18 and 35 years. The study participants visited the laboratory on three separate occasions, including a familiarization visit. Corticospinal responses were recorded during two conditions: (1) motor imagery of strong, forceful contractions of the hand and wrist flexors and (2) no motor imagery performed (i.e., control). Each participant underwent both conditions (i.e., one per visit). The order of the experimental conditions was randomized. All testing for each participant was separated by at least 24 h and occurred at the same time of the day (±one hour). This study was prospectively registered at ClinicalTrials.gov (Identifier: NCT03889548). The experimental design is illustrated in Figure 1.

### 2.2. Participants

A sample of 60 healthy adults were enrolled in this study. Of those enrolled, one elected not to return due to scheduling conflicts. Three participants were unresponsive to the TMS procedures at maximal intensities. Fifty-six healthy adults (25 males and 31 females; mean age ± SD = 22 ± 2 years) completed all study procedures and were included in the final analyses (Table 1). The inclusion criteria were healthy adults between 18 and 35 years of age with different screenings to qualify for each experimental group. Participants in the resistance–trained group had regularly participated in machine or barbell based resistance–training at least three (3) times per week over the previous six months. Participants in the untrained group had abstained from any resistance–training over the previous six months. The screening also ensured that all parties understood the procedures for the study and that no reason for exclusion was easily identified (e.g., existing comorbid conditions, medical issues that could interfere with data collection, or pose safety risks). In addition, participants completed a detailed in-house TMS screening questionnaire based on the recommendations described by Rossi et al. [17]. The major exclusion criteria for single-pulse TMS research includes a history of epilepsy, seizures, head trauma, and metal implants within the body.

### 2.3. Assessment of Resistance–Training Experience

Resistance–training experience was assessed via upper–body strength testing of 1RM bench press. Prior to maximal testing, participants performed several warm-up repetitions. The participants then performed 8–10 repetitions, with approximately 50% of their estimated 1RM. This was followed by another set of 3–5 repetitions of approximately 85% of the estimated 1RM. The barbell was then loaded with a weight near the participant’s estimated 1RM. Subsequently, they attempted a single repetition. After each successful attempt, the weight was progressively increased until the participant was no longer able to perform a repetition through the full range of motion. To ensure participant safety in the face of increasing muscular fatigue, no more than five 1RM attempts were allowed. At least three minutes of rest was given between attempts. The greatest load successfully lifted was recorded as the 1RM [18].

### 2.4. Grip Strength

Grip strength was assessed during the second visit to the laboratory. Hand grip strength was obtained via the use of a Jamar hand grip dynamometer (Jamar Technologies, Patterson Medical, Warrenville, IL, USA). The participants were seated with their elbow at 90° flexion and their wrist in a neutral position. They gripped the dynamometer and applied maximal grip strength for approximately five seconds. This was repeated three times for both the left and right hands, with 60 s of rest between trials.

### 2.5. Surface Electromyography (EMG)

During TMS testing, a wireless bipolar surface EMG sensor (interelectrode distance = 10 mm; Trigno EMG, Delsys, Inc., Natick, MA, USA) was placed over the belly of the non-dominant first dorsal interosseous (FDI) muscle, which was visually determined by asking the participants to abduct their index finger against light manual resistance. Prior to the placement of the sensor, the skin was shaved with a disposable razor, tape was used to remove dead skin cells and debris, and rubbing alcohol was used to clean the site. Following skin preparation, an EMG sensor was attached to the surface of the skin using adhesive tape. Prior to testing, the EMG signal was inspected to ensure low baseline noise (≤20 mV) and minimal line interference. EMG signal quality was monitored throughout the study, and additional skin preparation was performed as necessary.

### 2.6. TMS-Derived Corticospinal Excitability

Single-pulse TMS was performed using a MagStim 200^2^ stimulator (The Magstim, Whitland, UK). A 70-mm figure-of-eight focal coil was positioned tangential to the scalp, with the handle pointing backward and laterally at 45° from the midline. The “hot spot” was defined as the location over the motor cortex that elicited the largest peak-to-peak amplitude for FDI motor–evoked potentials (MEPs). Once identified, this location was marked on a Lycra^®^ cap to ensure consistent coil placement. Upon determining the hot spot, the resting motor threshold (RMT) was defined as the lowest stimulator intensity that could be used to reliably produce an MEP with a peak-to-peak amplitude ≥ 50 µV for five out of ten trials [19,20]. Once the hot spot and RMT were determined, six single TMS pulses were delivered to the hot spot at a stimulator intensity of 120% RMT before (pre) and after (post) the motor imagery intervention. Peak-to-peak amplitude of the MEPs was then determined (μV) and used as the primary dependent variable.

### 2.7. Motor Imagery

Immediately after the determination of RMT and pre-test measurements, the TMS administrator left the room to remain blinded to the treatment condition of the participant. At this time, the participant was given instructions regarding the motor imagery protocol by a separate investigator who was not blinded to the condition. During the motor imagery protocol, participants were instructed to close their eyes and imagine that they were maximally contracting the muscles in their hand and forearm, flexing their wrists, grabbing a heavy barbell, and maximally squeezing their hand. This was a kinesthetic imagery task in which participants were instructed to urge their muscles to contract maximally, as opposed to a simple visualization of performing the tasks [16].

After the reading of the motor imagery script was complete, the TMS administrator returned to perform the post-test measurements. The motor imagery task lasted throughout the duration of MEP amplitude posttesting. During the control condition, participants were instructed to sit relaxed with their eyes closed during testing. Upon completion of testing, the participants were instructed to open their eyes, and the motor imagery task was completed. The participants were seated with their hands placed on their lap in a relaxed fashion throughout testing. The total duration of the motor imagery task was approximately three minutes. 

### 2.8. Signal Processing

All TMS and EMG signals were acquired in sync with EMGWorks software (version 4.7.5, Delsys Inc., Natick, MA, USA). The EMG sensor bandwidth was 20–450 Hz, the input range was 11 mV, and the sampling rate was 1926 Hz. The peak-to-peak amplitude (mV) of each EMG signal during the TMS hotspot and RMT testing was quantified in real time. Peak-to-peak amplitude of EMG signals obtained during pre– and post– motor imagery TMS testing were processed using EMGWorks software (Delsys, Inc., Natick, MA, USA).

### 2.9. Statistical Analyses

Differences between resistance–trained versus untrained participants in 1RM bench press and grip strength relative to body mass (kg/kg) were characterized using independent sample *t*-tests, Cohen’s *d* effect sizes, and 95% confidence intervals (CI). MEP amplitude for the FDI served as the study’s primary dependent variable. Group (trained, untrained) served as a between-subjects factor, whereas time (pre, post) and condition (motor imagery, control) served as within-subject factors. To examine pre–to–post changes in MEP amplitude across groups and conditions, a three-way (group [trained, untrained] × condition [motor imagery, control] × time [pre, post]) mixed factorial analysis of variance (ANOVA) was conducted. In the event of a significant three-way or two-way interaction or main effects for group, condition, or time, Bonferroni-corrected pairwise comparisons were examined. An alpha level of 0.05 was used to determine statistically significant differences. In addition to null hypothesis significance tests, effect sizes via Cohen’s *d* statistics (for pairwise comparisons) and partial eta squared (η_p_^2^ for ANOVAs) were computed and evaluated. Small, medium, and large Cohen’s *d* values corresponded to 0.20, 0.50, and 0.80, respectively, whereas small, medium, and large partial eta squared values corresponded to 0.01, 0.06, and 0.14, respectively [21]. JASP software (version 0.16, The JASP Team, 2019) was used for all statistical analyses [22].

## 3. Results

The results of the independent sample *t*-tests indicated that the resistance–trained participants were significantly stronger for normalized 1RM bench press (trained mean ± SD = 0.922 ± 0.317, untrained = 0.566 ± 0.175 kg/kg; *p* < 0.001, *d* = 1.237, 95% CI = 0.182 to 0.528 kg/kg), but not grip strength (trained mean ± SD = 0.591 ± 0.121, untrained = 0.531 ± 0.073 kg/kg; *p* = 0.077, *d* = 0.543, 95% CI = −0.007 to 0.127 kg/kg).

**Figure 2:**
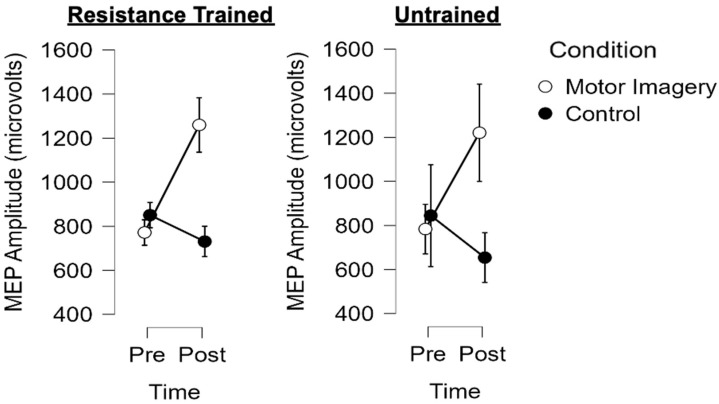
Mean ± standard error of the mean pre and post MEP amplitude for the resistance–trained (**left**) and untrained (**right**) participants for both motor imagery (white circles) and control (black circles) conditions. The results revealed no group differences (*p* > 0.05), but motor imagery increased MEP amplitude (see Figure 3).

**Figure 3:**
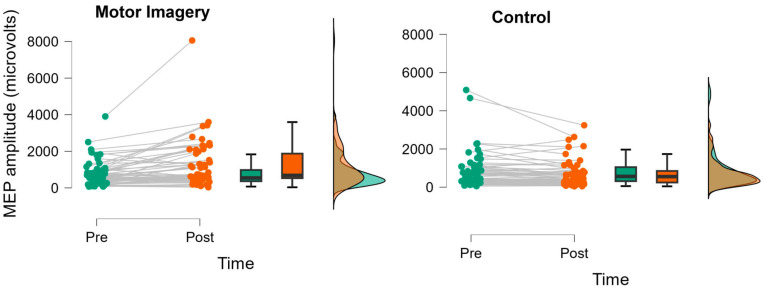
JASP raincloud plots displaying pre-post changes in MEP amplitude for motor imagery (**left**) and control (**right**) conditions. Based on the results of the three-way ANOVA, these data show responses collapsed across groups (*n* = 56). The results indicated that motor imagery significantly increased MEP amplitude, whereas no changes were observed in the control condition.

Figure 2 shows the mean ± standard error of the mean responses to the motor imagery and control conditions for both groups. The results of the three-way mixed factorial ANOVA indicated that there was not a significant group × condition × time interaction (*F* = 0.005, *p* = 0.946, η_p_^2^ < 0.001). In addition, there was not a significant interaction for group × time (*F* = 0.208, *p* = 0.650, η_p_^2^ = 0.004) or group × condition (*F* = 0.015, *p* = 0.902, η_p_^2^ < 0.001). There was also no main effect for group (*F* = 0.013, *p* = 0.911, η_p_^2^ < 0.001). There was, however, a significant condition × time interaction (*F* = 17.611, *p* < 0.001), with a very large effect size (η_p_^2^ = 0.243). The results from the Bonferroni-corrected pairwise comparisons indicated that motor imagery significantly increased corticospinal excitability (marginal means pre = 784.1 µV, post = 1246.6 µV; *p* < 0.001, *d* = 0.487), whereas a trivial decrease was observed for the control condition (pre = 854.0 µV, post = 698.9 µV; *p* = 0.728, *d* = 0.163). Figure 3 shows individual participant responses to the two conditions when collapsed across groups.

## 4. Discussion

Previous studies have demonstrated that motor imagery enhances strength and physical function via improvements in corticospinal excitability [15]. The extent to which resistance–training experience influences responsiveness to motor imagery has been unclear. The present study tested the hypothesis that participants with resistance–training experience would show greater acute increases in corticospinal excitability during a motor imagery task than those with no resistance–training experience. Our major finding was that motor imagery significantly increased corticospinal excitability. However, in contrast to our hypothesis, there were no differences between groups. These findings have important consequences for motor imagery prescription and suggest that motor imagery training may be equally beneficial for both resistance–trained and untrained populations. In the subsequent paragraphs, we address the implications of our work, its strengths and limitations, and propose ideas for future research.

The similar acute increases in corticospinal excitability observed in both groups suggests that resistance–training experience does not enhance the ability to engage cortical motor planning regions during imagery. We propose three ideas that may explain our findings. First, it is possible that overlapping cortical processes mediate both imagined and executed movements, such that physical resistance–training does not necessarily enhance motor imagery performance or excitability responses above untrained levels. Motor imagery relies predominantly on overlapping neural substrates involved in motor preparation [11,23,24]. Specifically, vividly imagining a movement engages the same frontal and parietal motor planning circuits as actually executing it [25,26]. While resistance–training develops cortical motor representations and muscle synergies for skilled movements [27], this motor memory is not spontaneously activated during isolated motor rehearsal without overt execution [24,25]. This explanation seems consistent with the work of Yoxon and Welsh [11], who concluded that imagined and executed motor tasks resulted in similar TMS-evoked thumb movements. A second, alternative explanation is that chronic exposure to resistance–training may exert a ceiling effect for neural adaptations, such that the performance of motor imagery may not increase corticospinal excitability to a greater extent than that observed in untrained participants. This theory is consistent with the time course of specific adaptations, with the early phases of resistance–training being largely controlled by neural adaptations [1,10]. A third and perhaps most relevant explanation is that the imagined task and assessment of corticospinal excitability for the FDI did not adequately reflect the training experience of the participants. While most adults perform resistance–training exercises that recruit multiple muscles and joints, we studied the FDI because of its ease of examination during TMS and the large body of literature studying hand muscles. As our inclusion criteria related to resistance–training were not specific to the hand or wrist, it is possible that the motor adaptations of our trained participants were not adequately evaluated. The notion that the specificity of training adaptations may have influenced our findings is supported by the discrepancy between our 1RM bench press versus grip strength data. Although our trained participants had significantly greater 1RM bench press than the untrained participants, the difference in grip strength between groups was only modest. Based on these findings, it is reasonable to speculate that a more task-specific TMS evaluation may have yielded different results.

While the results of our study indicate that resistance–training experience did not influence our main outcome, it is important to highlight that motor imagery increased corticospinal excitability by ~64%. Given the importance of corticospinal excitability for maximal force generation [28,29], our results provide further support for utilizing motor imagery as a means of facilitating neuromuscular adaptations in both trained and untrained participants. It should be noted, however, that our limited approach did not allow us to examine the underlying mechanisms of neuroplastic adaptations to motor imagery training. Therefore, we propose future research in this area to investigate the initial neural changes from a bout of motor imagery training and to examine if these responses are unique to trained versus untrained participants. For example, other research groups have experimented with kinesthetic imagery in combination with fMRI to understand the span of activation in different brain regions [30]. Once the initial changes are understood, future randomized control trials can examine the extent to which resistance–training combined with motor imagery enhances adaptation compared to either intervention alone. These types of studies will help practitioners and clinicians better target the nervous system when developing interventions.

This study has several strengths and limitations that should be acknowledged. In terms of the strengths of our approach, our design and controls were robust and included consistent personnel and a quiet, controlled laboratory environment. We also relied on well-established protocols for assessing RMT and corticospinal excitability of the FDI [19,20]. These strengths bolster the generalizability and confidence of our conclusions. However, several limitations should also be considered when placed in the context of the broader literature. First, our sample size was small. However, the observed effect sizes indicate that any differences between groups were small and inadequate statistical power was not a significant concern. Second, our participant characteristics evidently lacked an equal ratio between males and females, as well as between resistance–trained and untrained adults. The untrained group consisted of mostly females. While normalizing our 1RM and grip strength data to body mass may have minimized strength differences between sexes, it is possible that our findings may have been skewed by the disproportionate ratio of males and females between groups. While previous studies have attempted to compare corticospinal excitability between sexes [31,32], this concept should be investigated further. Third, our study design was acute, and there may have been more robust results if the intervention had been conducted over a longer period. Furthermore, it is important to note that we did not attempt to measure the efficacy of the motor imagery training. It is possible that the corticospinal responses reported herein varied as a result of the participants’ perceptions, vividness, and effort during data collection. Consequently, we cannot exclude the possibility that the effects of the motor imagery intervention may not have been due to the training at all. Indeed, several of our participants informally noted that they had difficulty with the motor imagery task. Although we encouraged participants to utilize a kinesthetic imagery approach, our results would have likely benefitted from attempts to capture their ability to perform kinesthetic motor imagery, such as via the kinesthetic vividness of imagery questionnaire [33]. Further, previous investigations have demonstrated improved cortical responses after motor imagery training [11,34]. However, in an attempt to utilize resistance–training experience as the primary moderating factor of imagery success, these considerations were outside the scope of this investigation. These limitations provide a plethora of opportunities for new research questions, including methodological investigations aimed at optimizing motor imagery protocols for resistance–trained versus untrained participants, as well as exploring the more widespread implementation of motor imagery in females, middle-aged and older adults, and those with neurological impairments undergoing exercise rehabilitation. As motor imagery has provided benefits in clinical populations, such as stroke patients and those recovering from severe injury [35], further knowledge of the brain’s response to motor imagery training might encourage exploration into advancing the field of injury recovery and improving the lives of the aging population.

## 5. Conclusions

In summary, while our findings provide further support for the efficacy of motor imagery to acutely enhance corticospinal excitability, these responses were similar for resistance–trained versus untrained participants. We suspect that our results may be related to overlapping cortical processes mediating both imagined and executed movements, a ceiling effect for neural adaptations in our resistance–trained sample, and/or a lack of task specificity for motor imagery relative to our participants’ resistance–training experiences. Although the strengths of our approach included a well-controlled and consistent laboratory environment and reliance on well-established protocols, a variety of minor limitations suggest that our work should be interpreted with caution. While we observed small effect sizes in our group comparisons, the robust impact of motor imagery on acutely increasing corticospinal excitability was encouraging. These concepts should continue to be explored in larger, more definitive research studies.

## Figures and Tables

**Table 1:** Participant demographics and relevant outcome measures. All measured values are expressed as mean ± standard deviation.

	All (*n* = 56)	Trained (*n* = 41)	Untrained (*n* = 15)
**Males (*n*, %)**	25 (44.6%)	21 (51.2%)	4 (26.7%)
**Females (*n*, %)**	31 (55.4%)	20 (48.8%)	11 (73.3%)
**Height (cm)**	171.0 ± 9.1	171.9 ± 8.7	168.5 ± 9.6
**Mass (kg)**	70.8 ± 15.0	72.1 ± 13.2	67.2 ± 18.7
***Outcome Measures***			
**1RM (kg)**	60.9 ± 30.9	68.5 ± 30.6	39.7 ± 20.1
**1RM/mass (kg/kg)**	0.828 ± 0.326	0.922 ± 0.317	0.566 ± 0.175
**Grip (kg)**	40.7 ± 11.6	42.6 ± 11.7	35.3 ± 9.3
**Grip/mass (kg/kg)**	0.575 ± 0.113	0.591 ± 0.1	0.531 ± 0.073
**MI RMT (%SO)**	55.0 ± 9.8	55.0 ± 9.0	57.0 ± 11.5
**Control RMT (%SO)**	56.0 ± 9.6	55.0 ± 9.4	56.0 ± 10.0

## Data Availability

The data presented in this study are available upon request from the corresponding author.
